# 3D planning and surgical navigation of clavicle osteosynthesis using adaptable patient-specific instruments

**DOI:** 10.1186/s13018-019-1151-8

**Published:** 2019-04-29

**Authors:** S. Roner, P. Bersier, P. Fürnstahl, L. Vlachopoulos, A. Schweizer, K. Wieser

**Affiliations:** 1 Computer Assisted Research and Development Group, Balgrist University Hospital, University of Zurich, Forchtrasse, 340 8008 Zurich, Switzerland; 2 Department of Orthopaedics, Balgrist University Hospital, University of Zurich, Zurich, Switzerland

**Keywords:** 3D surgical planning, Patient-specific instruments, Adaptable instruments, Compression guide, Surgical navigation, Length adaption

## Abstract

**Background:**

Preoperative three-dimensional planning and intraoperative navigation by patient-specific instruments is a promising method for the exact correction of bone deformities. Nevertheless, disadvantages of current concepts are the missing options of adapting the surgical plan intraoperatively. By providing the surgeons with a controlled length adjustment through the patient-specific instruments, the application area can usefully be expanded in the treatment of clavicle osteosyntheses.

**Methods:**

In three cases, preoperative three-dimensional surgical planning with the intraoperative use of patient-specific instruments was applied. The computer-assisted assessments of clavicle deformities, the preoperative plan, and the design of patient-specific instruments were created on the basis of computed tomography data. Reduction guides for restoring length and rotation according to the mirrored healthy contralateral side were enhanced with adaptable length adjustment functions. The screw thread of the reduction guides enabled temporary distraction of the clavicle fracture fragments and a controlled compression of the optionally used interposed bone block between clavicle fragments.

**Results:**

Navigated clavicle osteosyntheses by enhanced patient-specific instruments was executed uneventful in all three cases. The surgeon was able to adapt clavicle length in a planned axis intraoperatively as clinically desired.

**Conclusion:**

Computer-assisted planning of clavicle osteosynthesis and surgical navigation with additional adaptable patient-specific instruments can usefully expand the previous application areas. By using guided length adjustments, the fragments and optionally the graft can be compressed along a planned axis as desired to ensure optimal bone healing.

**Level of evidence:**

Basic science study, Surgical technique

**Electronic supplementary material:**

The online version of this article (10.1186/s13018-019-1151-8) contains supplementary material, which is available to authorized users.

## Introduction

Clavicle fractures are common, predominantly treated non-surgical and affected patients demonstrate pleasing outcomes [1]. In contrast, non- and malunions are rare but may result in reduced shoulder function and/or persisted pain [2, 3]. In symptomatic patients, surgical management is considered to improve shoulder function and reduce pain [4, 5].

Precise re-alignment and reconstruction of bone morphology are pursued by accurately reconstruct physiologic conditions [6]. To address bone resorption and shortening and/or to support bone healing, autogenous grafts from the iliac crest are frequently used [7]. Nevertheless, surgeries can be challenging and require exact planning and surgical execution [8]. Commonly, open reduction and internal fixation by osteosynthesis plate from superior is performed [4]. The aim is, according to the “Arbeitsgemeinschaft fuer Osteosynthesefragen” (AO) principles, to obtain fragment compression allowing optimal bone healing [4]. This can be ensured by eccentric drilling of the screw holes following application of compression through the plate or by temporally applied surgical instruments [9].

By recent developments in three-dimensional (3D) planning, more exact bone deformity analyses are enabled compared to conventional planning methods [10]. In particular, rotational deformities are analyzed more accurately in 3D compared to two-dimensional (2D) plain radiograph measurements [11]. Several studies demonstrated accurate execution of the surgical plan by 3D printed patient-specific instruments (PSI) in long bone deformities and in scaphoid non-unions [12–14]. In 2017, Vlachopoulos et al. described preoperative 3D planning and intraoperative navigation with patient-specific instruments as a feasible method for the treatment of midshaft clavicle malunions [15].

The rigid execution of a surgical plan by PSI limits the application of the method, for instance interventions needing a controlled compression between bone fragments or an adaption intraoperatively according to a 3D surgical plan. By providing the surgeon with a controlled length adjustment, and consequently a compression function in bone osteosynthesis, the application area of PSI can usefully be extended. Therefore, we hereby describe a guideline for performing clavicle osteosynthesis by adaptable 3D printed patient-specific instruments. By enabling a guided and modifiable distance between two clavicle fragments by a screw-system, optimal inter-fragment compression is ensured.

## Material and methods

### Data processing

Bilateral computer tomography (CT) images of the clavicle including the proximal part of the humerus and the entire scapula were acquired (slice thickness, 1 mm; 120 kV; Philips Brilliance 40 CT; Philips Healthcare, Eindhoven, The Netherlands). Segmentation of CT data followed by 3D bone model generation was performed with commercial software (Mimics, Materialize NZ, Leuven, Belgium). The preoperative planning software CASPA (Balgrist CARD AG, Zurich, Switzerland) and a commercial CAD Software (SolidWorks, Dassault Systèmes, Vélizy-Villacoublay, France) were used for computer-assisted preoperative planning and PSI design [16].

### Deformity analysis and surgical planning

The healthy contralateral clavicle was mirrored and served as reconstruction template [17]. By iterative closest point (ICP) algorithm, the mirrored bone model was superimposed on the medial fragment of the pathological clavicle as previously described [16]. Subsequently, the lateral fragment was aligned with the template to calculate the desired transformation. Due to excessive shortening, this might result in a large gap between the two fragments. By considering the history of the patient and the deformity analysis, a surgical plan was established according to the surgeon’s decision whether or not to use iliac bone autogenous graft. In either case, the surgeon balanced the preoperative plan between a complete length restoration of the clavicle and a reduction of the gap size between the fragments. Optionally, references lengths to use intraoperatively were defined. Excluding the 3D planning, the production costs of the patient-specific instruments are estimated to be around 600 Euro.

**Fig. 1:**
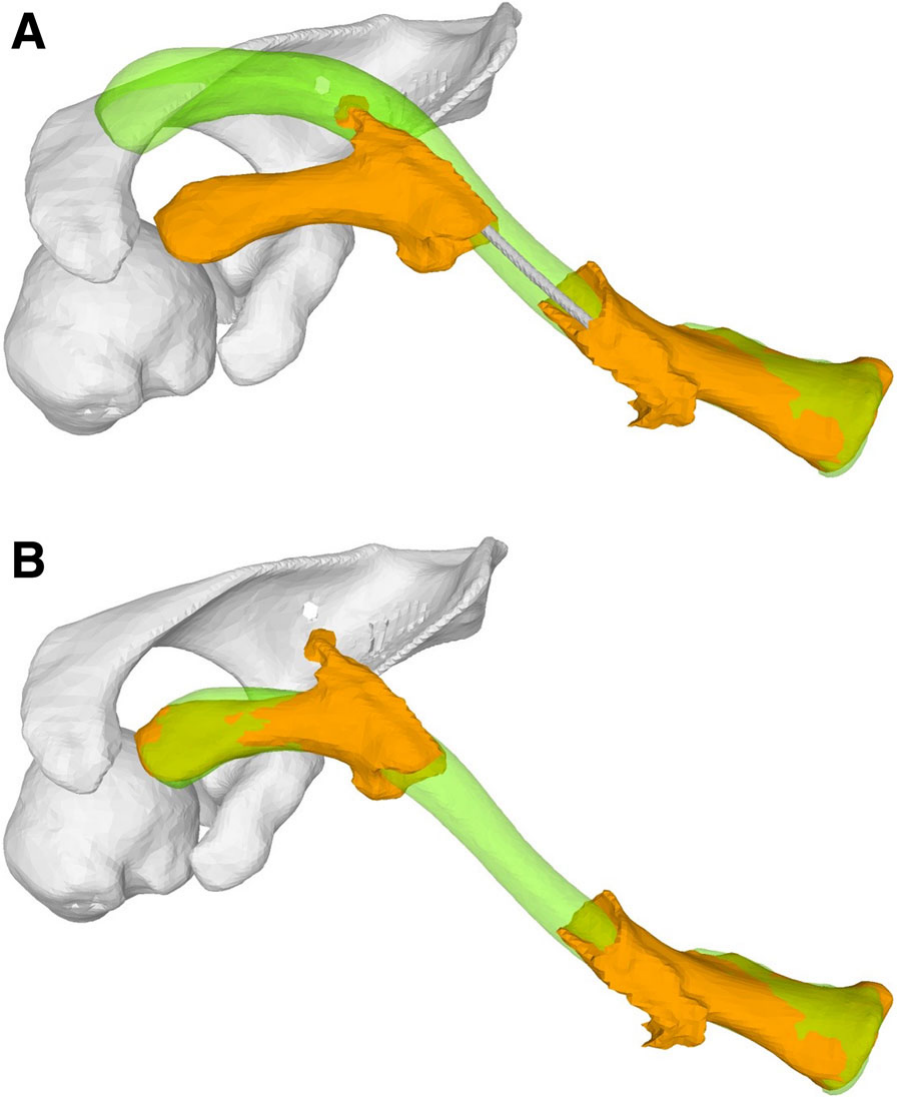
**a** Case 1 with clavicle non-union and the intramedullary nail**. b** Preoperative plan with alignment to the mirrored contralateral site

**Fig. 2:**
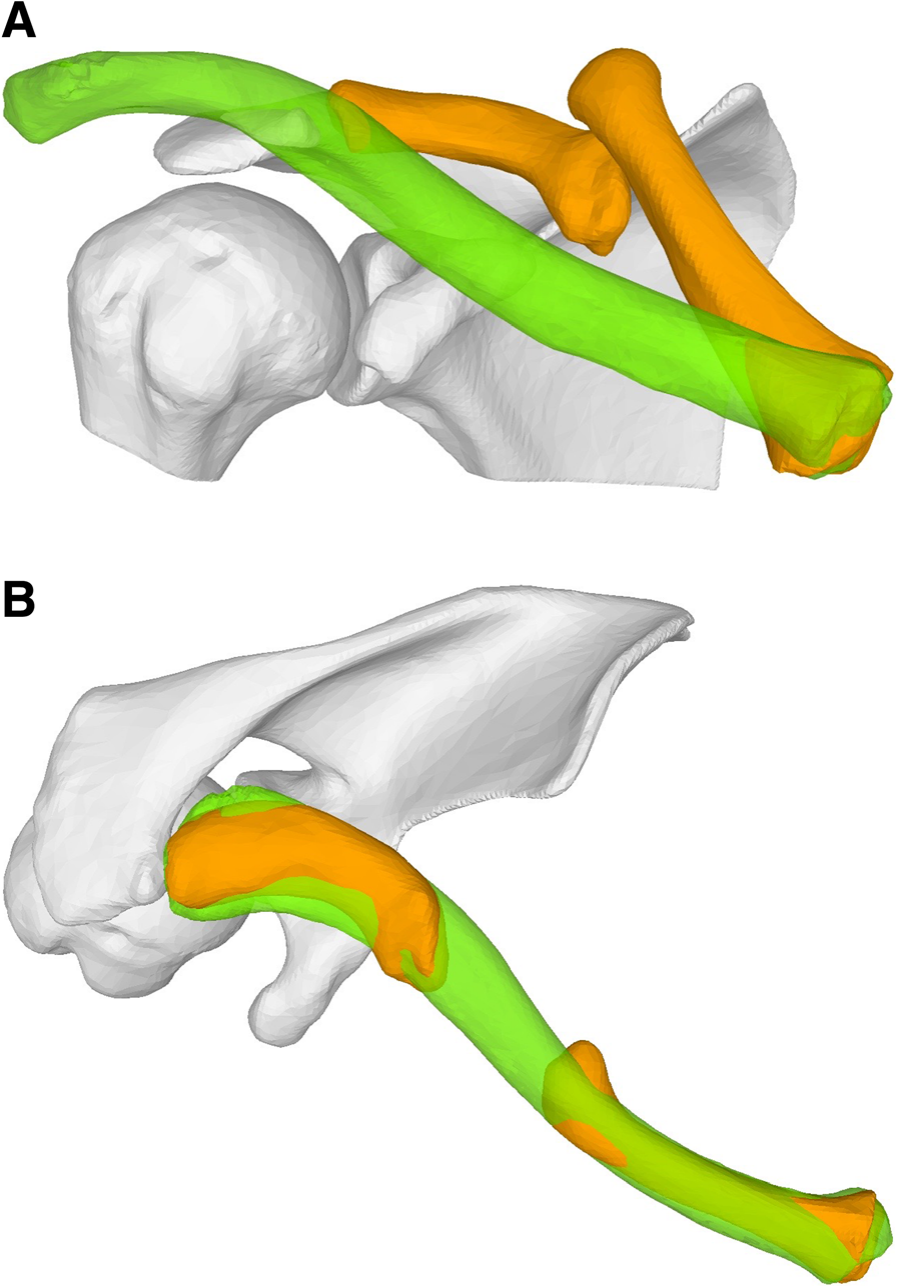
**a** Case 2 with dislocated clavicle non-union**. b** Preoperative plan with alignment to the mirrored contralateral site

**Fig. 3:**
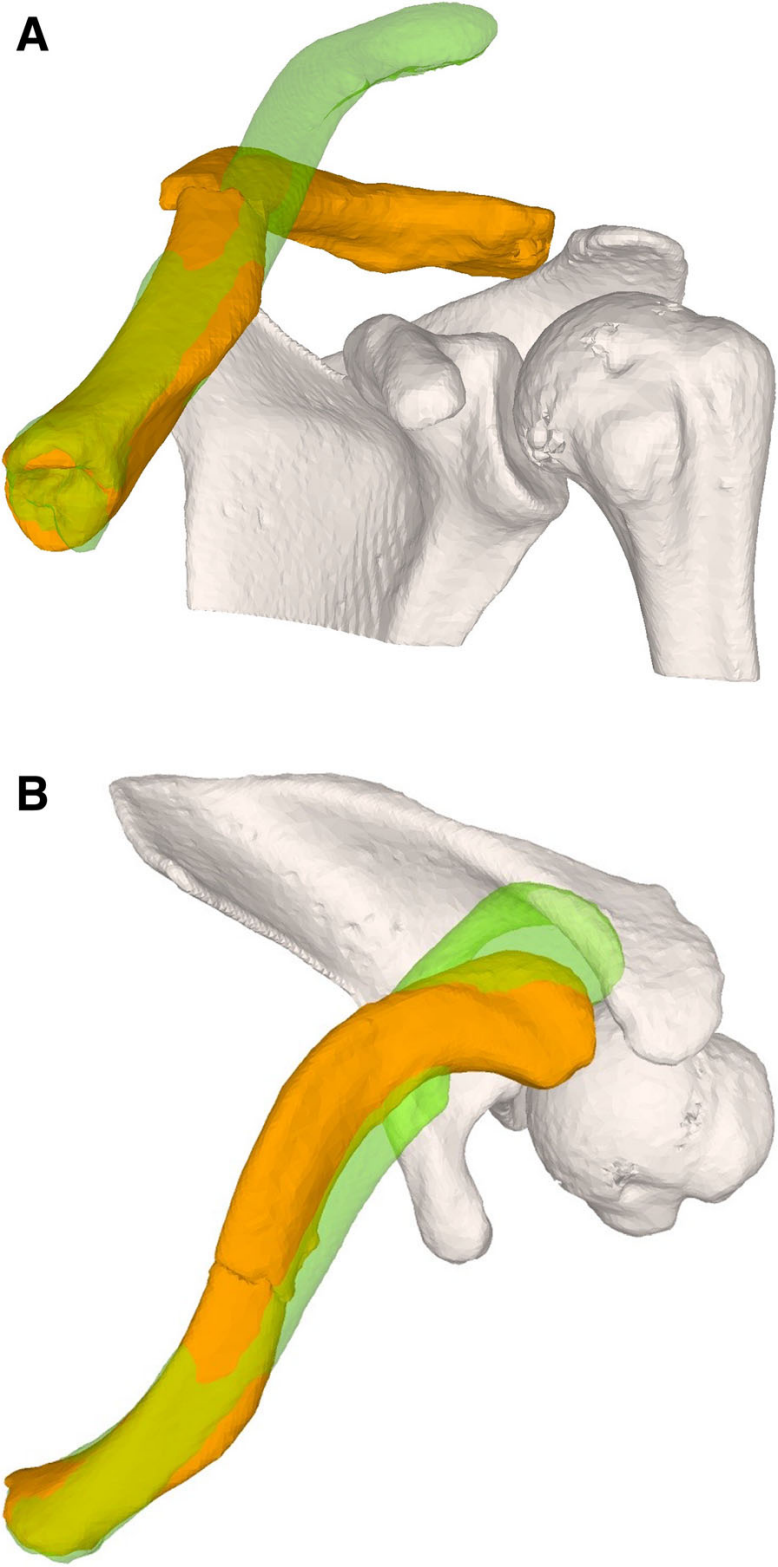
**a** Case 3 with re-fractured clavicle malunion**. b** Preoperative plan with fracture reduction and superimposed contralateral clavicle

In case 1, a 54-year-old female patient presented with clavicle non-union following several previous osteosyntheses of a mid-shaft fracture. Because of a bone gap of 4 cm, the surgeon was planning an osteosynthesis with interposition of an iliac bone autogenous graft (Fig. 1). In case 2, a 17-year-old male patient presented with a dislocated non-union following a mid-shaft fracture (Fig. 2). Similar to the first case, a bone gap of 3 cm was created after the planned reduction of the clavicle. The third case was a 16-year-old male with re-fracture of a malunited clavicle 4 years after the first fracture of the left clavicle. The reduction of the fracture demonstrated a residual malunion of the clavicle compared to the contralateral site (Fig. 3). Since the patient had no complaints before the re-fracture of the clavicle, the surgeon decided to reduce the fracture without re-alignment to the contralateral site, avoiding an additional osteotomy or the need of structural iliac crest bone grafting of this acute fracture.

## Guide design

### Basic guides

**Fig. 4:**
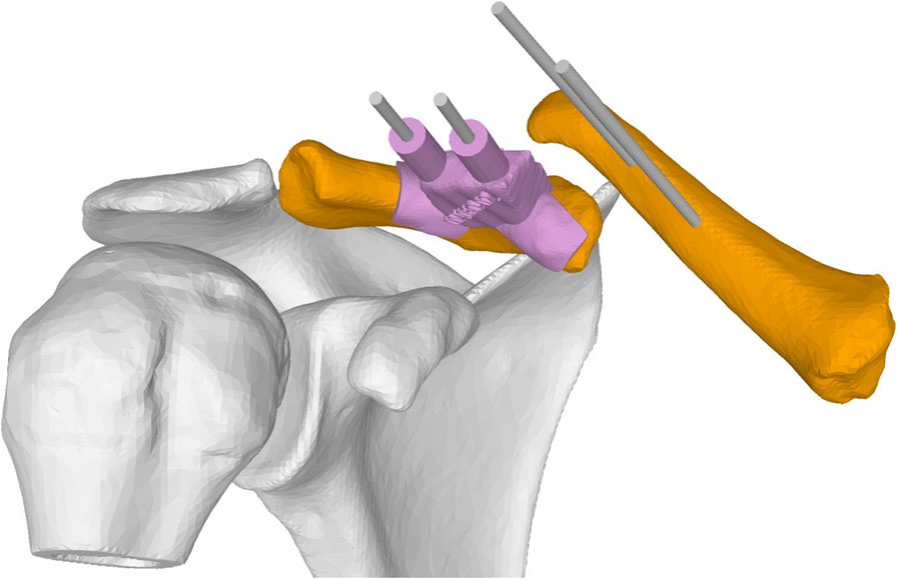
Design of patient-specific base guide on lateral fragment

The undersurface of the basic guide is molded as a negative of the bone surface ensuring correct positioning in the surgery. Because of the oval morphology of the clavicle shaft fragments, the intraoperative identification of the correct position is straightforward in all but the medial/lateral direction. For this reason, the guides are designed considering the fracture line as a characteristic landmark. By overlapping the surface negative into the superior-anterior non-union/fracture site, the base guide position is perfectly defined (Fig. 4). Once the correct guide positions are applied, references K-wires (2–2.5 mm) are set through the drill sleeves integrated into the basic guides of each fragment. The sets of K-wires are then used for navigating fragment reduction and as reference K-wires for the following reduction-guide. The directions of these K-wires in their preoperative configurations were computed such that the medial K-wire pair and the lateral K-wire pair are parallel to each other with the post-reduction position. A detailed description of the guide design principles was previously published in the reconstruction of scaphoid non-unions [18].

### Reduction—compression guide

The first parameter to be determined is the axis of the length adaption. The direction is manually defined with the axis representing a line between the ends of the fragments. The graft is clamped between the fragments when applying a perpendicular compression force; consequently, a displacement can be minimized.

**Fig. 5:**
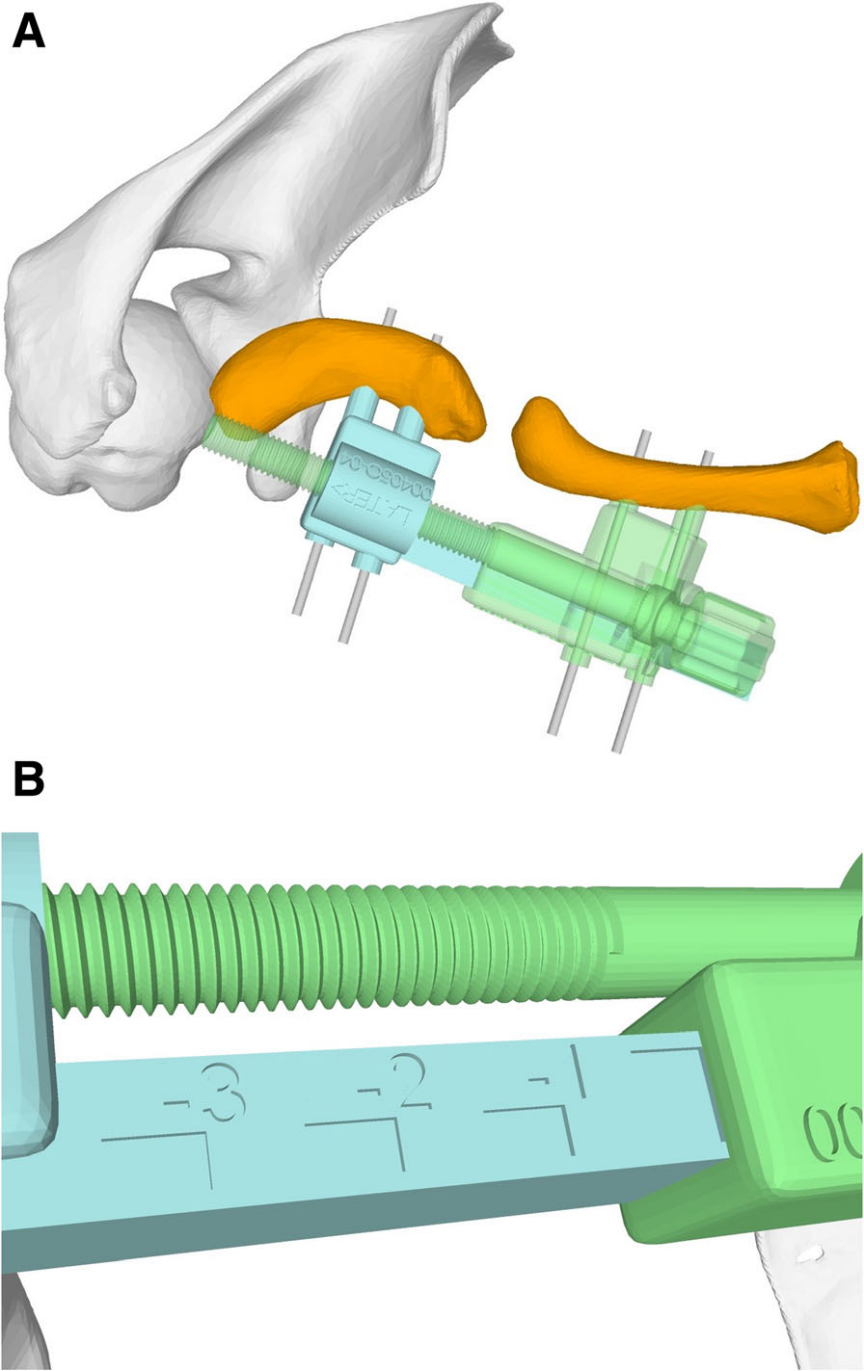
**a** Patient-specific reduction guide enabling a length adaption along a planned axis. **b** Guide bar allowing intraoperative feedback

Once the length adaption axis is defined, the basic construction of the reduction guide allows constraining all degrees of freedom except the translation along the planned axis. By a parallel screw on the opposite side of the reference K-wires, a controlled length adaption is enabled. The result is a two-part guide, with one part containing the male part of the linear guidance and the female thread for the screw and the other part the female part of the guidance and the screw (Fig. 5a). The part of the guide containing the screw is designed to allow the screw to drive according to a guide bar (Fig. 5b). The length adaption can be used to pull both fragments apart as well as to apply compression between the fragments or on the iliac crest graft.

### Surgical technique

**Fig. 6:**
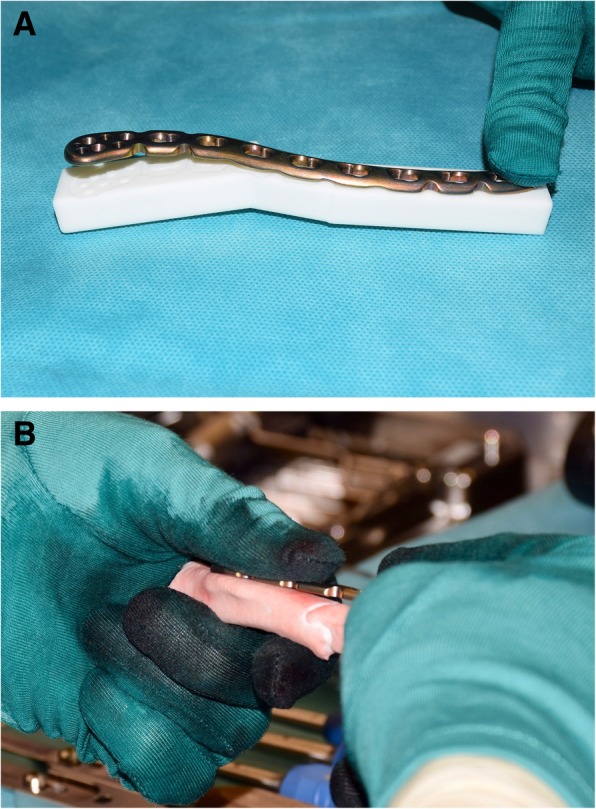
Pre-bending of osteosynthesis plate on 3D printed bone models in case 2 (**a**) and case 3 (**b**)

**Fig. 7:**
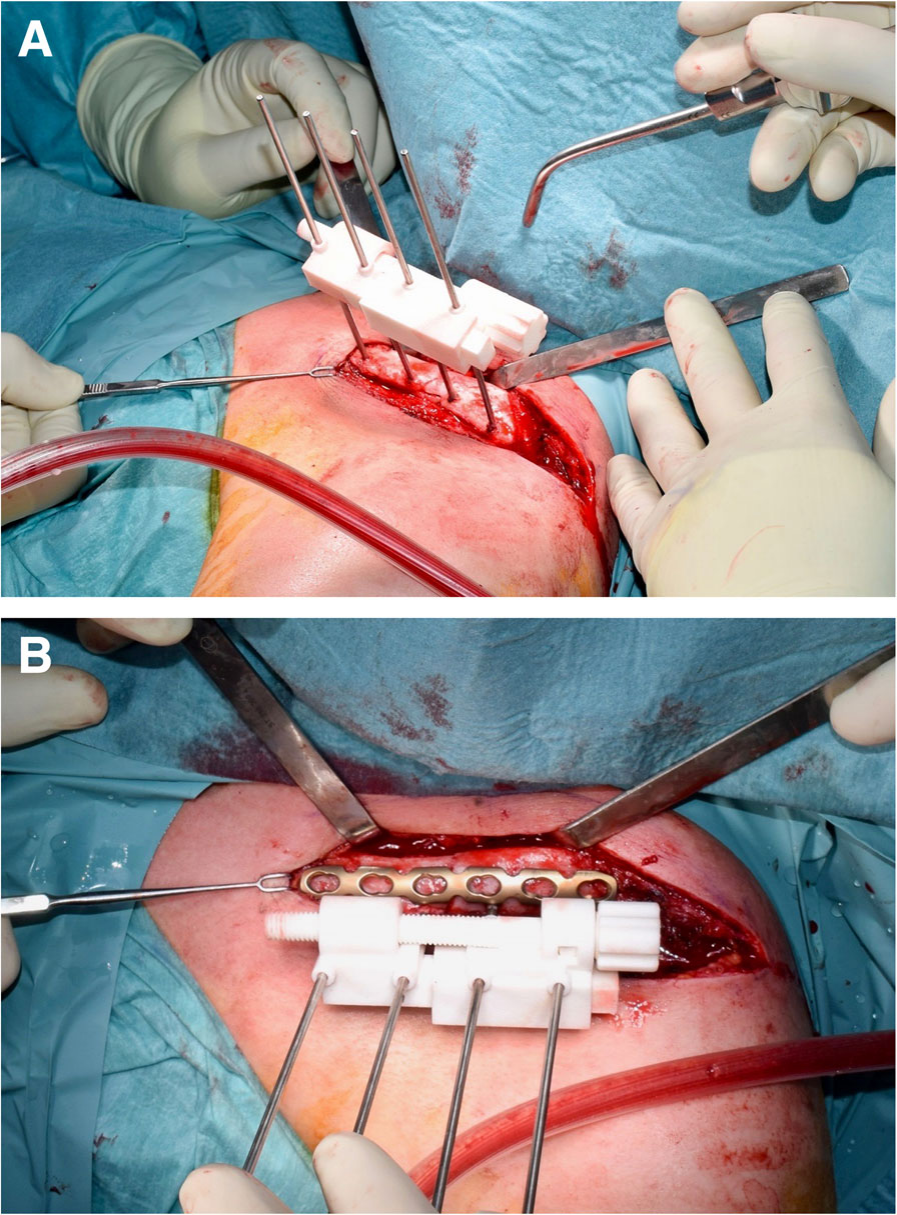
**a** Intraoperative situ of case 3 with application of the reduction-guide**. b** Temporary fixation by reduction guide and osteosynthesis plate positioning


A 3D printed bone model of the planned clavicle reduction was optionally used to pre-bend the osteosynthesis plate (3.5 mm LCP Superior Anterior Clavicle Plates, Synthes, Switzerland) outside of the situs (Fig. 6a, b). An anterior approach to the clavicle was conducted to visualize the fragments in preparation of the osteosynthesis. After denuding the bone surface area to apply the base guide, two K-wires were applied on each the medial and lateral fragment anterior-superiorly. Subsequently, the guides were removed and the assembled reduction-compression guide was inserted over all four K-wires (Fig. 7a). By setting a slightly longer length than planned, the reduction of the lateral fragment can be facilitated and/or can help to optionally insert the harvested and preshaped (according to the preoperative planning) iliac crest bone graft. Subsequently, compression between the lateral and medial fragment was conducted. The pre-bent plate was placed superior on the clavicle and fixated by standard cortical bone and/or locking screws as desired. An image intensifier confirmed plate position and accurate screw length. Finally, the four K-wires and the compression PSI were removed and the wound was closed. An illustration of the principles of the hereby described surgical technique is provided in the included (Additional file 1).

Additional file 1: Principles of the clavicle reduction by adaptable patient-specific instruments. (MP2 31441 kb)


## Results

**Fig. 8:**
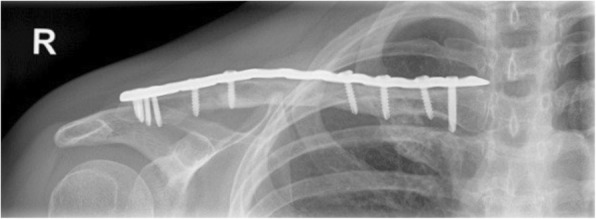
Postoperative AP X-ray of the clavicle in case 1

The navigated clavicle osteosynthesis was executed uneventful in all three cases. Bone consolidation was observed after 8–12 weeks in plain radiographs (Fig. 8). All three patients were symptom-free and returned to their daily activities no later than 12 weeks postoperative.

Length of the corrected clavicle in an AP plane was in the first case 94.4% of the contralateral template and in the second case 96.6%, respectively. In case 3, the re-fractured malunion was aligned with compressive bone contact and therefore no length adaption but a compressive force between the fragments was executed.

## Discussion

We hereby demonstrate the adaption of a surgical navigation intraoperatively according to a 3D plan as a promising method in the treatment of non-unions and re-fractures of previous malunited clavicles. To our knowledge, this is the first description of a patient-specific 3D planned compressive guide. By using the described length adjustment functionality of the reduction PSI, a compression force can optimally be applied between two clavicle fragments. The amount of length adjustment is quantified by a measuring scale on the reduction guide, providing a valuable feedback intraoperatively. Surgical challenges like the controlled insertion of the bone graft can be facilitated by temporarily increasing the distance between the fragments and soft tissue expanding can be eased. Furthermore, a stable temporarily fixed reduction is the prerequisite for the easy application of pre-bended plates using cortical bone and/or locking-screw system [19].

Recent publications showed the advantage of 3D planning and the intraoperative use of PSI to optimize the outcome of operative treatment of clavicle mal- and non-unions [16, 20]. Vlachopoulos demonstrated the benefit and versatility of computer-assisted planning and navigation of corrective osteotomies of the midshaft clavicle [16]. However, the drill-sleeves incorporated in the reduction guide had to be removed on one fragment site and the reduction was fixated temporally with a bone-holding force to achieve the compression through the navigated inter-fragmentary screw. In 2018, Grewal et al. demonstrated two cases of corrective osteotomies in symptomatic clavicular malunion using prereduction and reduction guides and recommend the technique in complex cases [20].

The benefit of 3D planning provides visualization of several surgical solutions, e.g., using iliac crest versus solely realignment clavicular fragments. By the means of 3D planning, the exact graft size can be measured and, consequently, a patient-specific guide for exact graft harvesting can additionally be helpful. The surgical plan can be used for selecting the optimal plate size and position. Furthermore, 3D printed bone models can be used as a template for pre-bending the osteosynthesis plate [21]. As a result, this improved preoperative planning can reduce the time of surgery [22]. While this technique was described hereby for the osteosynthesis of clavicles, the principle might also be applicable for further anatomies. In our opinion, the extra expenses are sensibly used considering the difficulty and risks of the intervention in selected cases.

So far, a limitation of the presented technique is that adaption of the surgical plan is only possible in one axis. In the future, we intend to optimize the possibilities of the hereby-described technique and extend the application area of adaptable PSI. However, too much intraoperative freedom might on the other side increase potential wrong execution of the preoperative plan. For stability reasons, the described technique uses quite cumbersome instruments, resulting in possible interference and reduction of visualization of the surgeon during plate application. Moreover, studies investigating outcome compared to state-of-the-art techniques need to be performed in larger clinical trials to compare surgical techniques

## Conclusion

Intraoperative navigation with adaption according to a 3D surgical plan is enabled with the hereby proposed technique of enhanced patient-specific instruments. Thereby, fragment compression in clavicle (re-)osteosynthesis can be applied allowing optimal fragment healing.

## Abbreviations

2D: Two-dimensional
3D: Three-dimensional
CT: Computed tomography
PSI: Patient-specific Instruments
